# Endocrine nanozymology: Nanozyme applications in diabetes, obesity, and hormonal disorders

**DOI:** 10.7150/thno.122577

**Published:** 2026-01-01

**Authors:** Huilun Lu, Zizhong Liu, Ya-chao Wang, Eudald Casals, Felicia A Hanzu, Manuel Morales-Ruiz, Muling Zeng, Gregori Casals

**Affiliations:** 1 Department of Geriatrics, Shenzhen Longgang Second People's Hospital, Shenzhen, Guangdong, China.; 2 Internal Medicine Ward I, Shenzhen Longgang Second People's Hospital, Shenzhen, Guangdong, China.; 3 Department of Neurosurgery, Institute of Translational Medicine, Shenzhen Second People's Hospital/The First Affiliated Hospital of Shenzhen University, Shenzhen, China.; 4 Premium Research SL, 19003, Guadalajara, Spain.; 5 Endocrine Disorders Group, Institut d'Investigacions Biomèdiques August Pi i Sunyer (IDIBAPS)/ Hospital Clínic, Barcelona, Spain.; 6 Endocrinology Department, Hospital Clínic, Barcelona, Spain.; 7 Centro de Investigación Biomédica en Red de Diabetes y Enfermedades Metabólicas Asociadas (CIBERDEM), Spain.; 8 Department of Biochemistry and Molecular Genetics, Hospital Clínic, FRCB-IDIBAPS, Barcelona, Spain.; 9 Centro de Investigación Biomédica en Red de Enfermedades Hepáticas y Digestivas (CIBEREHD), Spain.; 10 Department of Biomedicine, Faculty of Medicine and Health Science, University of Barcelona, 08036 Barcelona, Spain.; 11 Institut de Ciència de Materials de Barcelona, ICMAB (CSIC), Campus UAB, Bellaterra, Spain.; 12 Department of Fundamental and Clinical Nursing, Faculty of Nursing, University of Barcelona, L'Hospitalet de Llobregat, 08907 Barcelona, Spain.

**Keywords:** nanozymes, *diabetes mellitus*, thyroid, adrenal, metal-organic frameworks

## Abstract

Nanozymes, engineered nanomaterials with enzyme-like catalytic activity, are emerging as versatile tools in biomedicine due to their catalytic tunability and higher chemical, thermal, and structural stability compared to natural enzymes. While widely studied in oncology and inflammation, their potential in endocrine disorders remains comparatively underexplored due to the historical focus of nanomedicine on cancer-related oxidative stress, the complexity and heterogeneity of endocrine signaling networks that hinder direct translation, and the scarcity of preclinical models that capture the dynamic and systemic nature of endocrine physiology. However, disruption of hormonal homeostasis by free radical imbalance points to the significant potential of nanozymes in endocrine disorders. By mimicking redox-active enzymes such as catalase, superoxide dismutase, and peroxidase, nanozymes regulate reactive oxygen species (ROS), thereby influencing hormone biosynthesis, receptor sensitivity, and redox signaling. They also offer advantages such as composite architectures, targeted delivery, and integration into smart platforms like hydrogels and biosensors. This review explores the expanding role of nanozymes in endocrine and metabolic diseases, including diabetes, obesity, thyroid and adrenal dysfunctions, and reproductive disorders. We highlight advances in glucose biosensing, hormone detection, redox-targeted therapies, and regenerative approaches. Despite promising preclinical data, there is a lack of clinical trials and long-term biosafety assessments of nanozymes, underscoring the need for further translational studies. By bridging nanotechnology and hormonal regulation, we outline future research directions toward integrating nanozymes into endocrine diagnostics and therapeutics.

## 1. Nanozymes in biomedicine: catalytic properties and classifications

The advent of nanotechnology has led to the emergence of nanozymes (NZs), a class of nanomaterials that mimic the catalytic activities of natural enzymes. Since their conceptual introduction in 2004 and the first demonstration of intrinsic enzyme-like activity in ferromagnetic nanoparticles in 2007 1, NZs have attracted widespread attention for their robust catalytic activity, physicochemical stability, and cost-effectiveness. Unlike natural enzymes, which are often susceptible to environmental conditions such as temperature and pH, NZs provide a stable alternative for various catalytic processes, particularly in biomedical settings. Their multifunctionality and tunability have enabled their integration into fields ranging from biosensing and diagnostics to targeted therapy and imaging.

NZs are generally classified based on their elemental composition and structure, with each category possessing unique catalytic properties and mechanisms. The major categories include metal-based, carbon-based, metal sulfide-based, rare-earth element-based, and hybrid NZs. Table 1 summarizes their catalytic activities, key advantages, and associated risks.

Metal-based NZs are among the most extensively studied due to their diverse electronic configurations and redox potential. Iron oxide nanozymes (Fe_3_O_4_NZs), for instance, exhibit peroxidase-like activity by catalyzing the decomposition of hydrogen peroxide (H_2_O_2_) into reactive oxygen species (ROS), a property that has proven valuable in inducing oxidative stress in tumor microenvironments 1,2. The mechanism involves the redox cycling between Fe^2+^ and Fe^3+^ states, which facilitates electron transfer during catalytic reactions. Gold nanozymes (AuNZs) present oxidase-like activity, which enables them to catalyze oxidation reactions without the need for H_2_O_2_, making them particularly suitable for biosensing and colorimetric assays 3. The mechanism of action in AuNZs is influenced by their surface plasmon resonance and electron-rich surface. Cerium oxide nanozymes (CeO_2_NZs) mimic both catalase and superoxide dismutase (SOD) activities, attributed to the redox cycling between Ce^3+^ and Ce^4+^. This dual functionality allows CeO_2_NZs to scavenge ROS and reduce oxidative damage in neurodegenerative diseases and inflammatory conditions 4. Copper oxide nanozymes (CuONZs) possess both oxidase and peroxidase-like activities. Their efficacy in antimicrobial applications stems from their ability to generate ROS and disrupt bacterial membranes 5.

Carbon-based NZs include materials such as graphene oxide and carbon nanotubes. Graphene oxide displays peroxidase- and oxidase-like activities due to its high surface area and electronic conductivity. These properties enhance their catalytic efficiency in redox reactions, especially in biosensing and environmental remediation 6. Carbon nanotubes, owing to their tubular morphology and high aspect ratio, facilitate electron transport, thereby enhancing catalytic performance. Their application ranges from electrochemical sensors to nanocarriers for drug delivery 7.

Metal sulfide NZs are an emerging class of materials known for their semiconducting properties. Cadmium sulfide nanozymes (CdSNZs) exhibit light-dependent photocatalytic activity. Under visible light irradiation, CdSNZs can generate ROS through photoinduced electron-hole pair formation, making them effective agents in photodynamic therapy 8. Molybdenum disulfide nanozymes (MoS_2_NZs), on the other hand, possess intrinsic peroxidase-like activity. Their two-dimensional layered structure and sulfur-rich surface facilitate catalytic decomposition of H_2_O_2_, enabling their application in biosensors and tumor therapy 9.

Rare-earth elements, such as lanthanum and europium, form NZs with distinctive electronic properties. Lanthanum-based NZs exhibit catalytic activity through electron transfer mechanisms, contributing to applications in medical imaging and targeted therapy 10,11. Europium-containing nanoparticles are used in luminescent biosensing due to their strong photoluminescent properties, driven by intra-4f transitions. These materials can serve dual functions in diagnostics and imaging-guided therapy 11.

To enhance functionality, researchers have developed hybrid NZs that combine multiple catalytic centers or integrate organic and inorganic components. Metal-organic frameworks (MOFs), composed of metal nodes and organic linkers, offer tunable structures with intrinsic enzyme-like activity. Their porous nature and catalytic versatility make them suitable for applications in drug delivery and pollutant degradation 12. Core-shell structured NZs, which include a catalytically active core surrounded by a protective or functional shell, allow for improved stability, biocompatibility, and targeted activity. These structures are particularly useful in theranostics, combining therapeutic and diagnostic functions in a single platform 13.

## 2. Diagnostic and therapeutic applications of nanozymes. Advancing toward endocrine applications

### 2.1. Main applications: from cancer to biosensing

The biomedical applications of NZs span a diverse spectrum of therapeutic and diagnostic fields. In cancer therapy, for instance, NZs have been harnessed to exploit the elevated oxidative stress within tumor microenvironments. Fe_3_O_4_ and CeO_2_ NZs are of particular interest in this regard. These nanomaterials catalyze the decomposition of H_2_O_2_, which is abundantly present in tumors, to generate ROS, causing selective oxidative damage in cancer cells without affecting the healthy tissues.

This selective cytotoxicity, combined with the potential to engineer surface functionalities for targeted delivery, positions NZs as powerful tools for precision oncology 2,4,14.

Beyond oncology, NZs show promise in neurodegenerative diseases, although more research is needed to confirm their ability to cross the blood-brain barrier. In Alzheimer's and Parkinson's disease, elevated ROS leads to neuronal damage. CeO_2_NZs, through their capacity to scavenge superoxide and H_2_O_2_ through Ce^3+^/Ce^4+^ redox cycling, offer neuroprotective benefits by maintaining redox homeostasis and reducing neuroinflammation 15.

Infectious diseases and wound healing also constitute a vital application domain for NZs. CuO and other transition metal-based NZs have emerged as potent antibacterial agents, primarily through their ability to generate ROS and disrupt microbial membranes. These NZs do not rely on traditional antibiotic pathways, thereby reducing risk of resistance. They can be embedded in hydrogels or device coatings to create antimicrobial environments 5,16.

NZs have also revolutionized biosensing and diagnostics. Their intrinsic catalytic activity enables signal amplification, allowing for the detection of biomarkers at ultra-low concentrations. Au NZs, with their excellent oxidase-like activity and ease of functionalization, are extensively used in colorimetric and electrochemical biosensors for the rapid detection of glucose, cholesterol, pathogens, and tumor markers in clinical samples. Their adaptability and robustness make them promising candidates for point-of-care and wearable biosensing technologies 3,17.

In addition to therapy and diagnostics, NZs play a pivotal role in bioimaging and theranostics. Lanthanide-doped and europium-based NZs possess unique luminescent properties that enable high-resolution imaging. When integrated with magnetic or catalytic functionalities, these NZs can simultaneously facilitate imaging and treatment, allowing for real-time monitoring of therapeutic responses. For instance, gadolinium-doped NZs support both MRI imaging and therapeutic modulation 11,18.

### 2.2. Endocrine nanozymology: a new frontier in hormonal disease management

As shown, NZs offer innovative and highly adaptable solutions across multiple biomedical domains. Their enzyme-mimetic activities, combined with superior stability, cost-efficiency, and functional tunability, address long-standing limitations of natural enzymes and unlock new possibilities in diagnostics, imaging, and therapeutics. Of particular interest is the growing body of evidence pointing to the applicability of NZs in the context of metabolic and endocrine disorders. In recent years, an increasing number of studies have begun to explore the role of NZs in the diagnosis and management of *diabetes mellitus* and other endocrine dysfunctions. Despite this emerging potential, NZs remain underutilized in endocrinology, due not only to the historical focus of nanomedicine on cancer, but more importantly to the inherent complexity of hormonal signaling. Endocrine disorders, by their very nature, are systemic diseases that affect multiple organs through hormonal dysregulation, a distinctive feature that sets them apart from other medical disciplines. This systemic reach, combined with the dynamic and heterogeneous nature of endocrine networks, presents unique challenges for diagnosis and treatment. These factors justify the emergence of endocrine nanozymology as a distinct interdisciplinary field, integrating nanotechnology and enzymology to address the systemic complexity of endocrine dysfunctions.

Unlike previous reviews that primarily focus on oncology or general biosensing applications, this work provides a comprehensive and integrative overview of NZ applications specifically in endocrine disorders, including underexplored areas such as circadian rhythm regulation and hormone-responsive theranostic platforms. By providing an integrated perspective on their catalytic properties and biomedical utility, the following sections aim to highlight not only the present achievements of NZ research but also its promising future in the diagnosis, monitoring, and treatment of endocrine-related diseases. While the core applications of this research focus on conditions such as diabetes, atherosclerosis, and obesity, emerging areas of interest include thyroid, adrenal, and chromaffin tumors. Notably, many of these endocrine disorders disproportionately affect women, emphasizing the potential of nanozyme-based innovations to help address gender-related disparities in diagnosis and care.

## 3. Translational hurdles of nanozymes

Despite their promise, the transition from promising laboratory findings to clinically viable solutions remains hindered by several unresolved scientific and regulatory challenges.

*Toxicity and biocompatibility*. NZs can induce oxidative stress, inflammation, and cytotoxicity due to their high surface reactivity and catalytic activity. For example, iron oxide and copper-based nanozymes generate ROS, which can damage cellular components 19. The shape, charge, and surface chemistry of NZs influence their interaction with biological systems, potentially triggering immune responses or membrane disruption 20. Surface modifications, such as PEGylation or biomimetic coatings, have been shown to reduce toxicity and improve circulation times 21.

*Biodistribution and clearance*. NZs often accumulate in non-target organs like the liver, spleen, and kidneys due to uptake by the reticuloendothelial system, raising concerns about long-term safety 21. Their clearance is influenced by size, surface charge, and protein corona formation. Inter-individual variability in metabolism and immune response further complicates pharmacokinetics, necessitating personalized design strategies 22.

*Synthesis, scalability, and reproducibility*. While NZs can be synthesized via various chemical and physical methods, scaling up production while maintaining consistent catalytic activity and morphology remains a challenge 20. Batch-to-batch variability affects therapeutic efficacy and safety. Good Manufacturing Practice (GMP) compliance requires stringent control over particle size, shape, and surface functionalization, which is not yet standardized for NZs 21.

*Regulatory ambiguity*. NZs occupy a regulatory gray zone, often falling between drugs, biologics, and devices. Agencies like the FDA and EMA lack specific guidelines for NZ evaluation, delaying clinical translation 19. The absence of harmonized international standards complicates toxicity testing, quality control, and approval pathways 23.

*Mechanistic uncertainty and predictive modeling*. Unlike natural enzymes, NZs may exhibit multi-modal or context-dependent catalytic behaviors. Their activity can be influenced by environmental factors such as pH, temperature, and macromolecular crowding 22. This complexity hampers mechanistic understanding and predictive modeling. Emerging tools like machine learning and systems toxicology are being explored to forecast nanozyme behavior and optimize design 24.

*Environmental and long-term safety*. Concerns about environmental persistence and bioaccumulation of NZs are growing. Their long-term effects on ecosystems and human health remain poorly understood. Studies suggest that chronic exposure could lead to epigenetic changes, lysosomal destabilization, and immunogenicity 23. Comprehensive life-cycle assessments and environmental impact studies are urgently needed to ensure biosafety 25.

## 4. *Diabetes Mellitus*, insulin resistance, obesity, and inflammation

**Figure 1:**
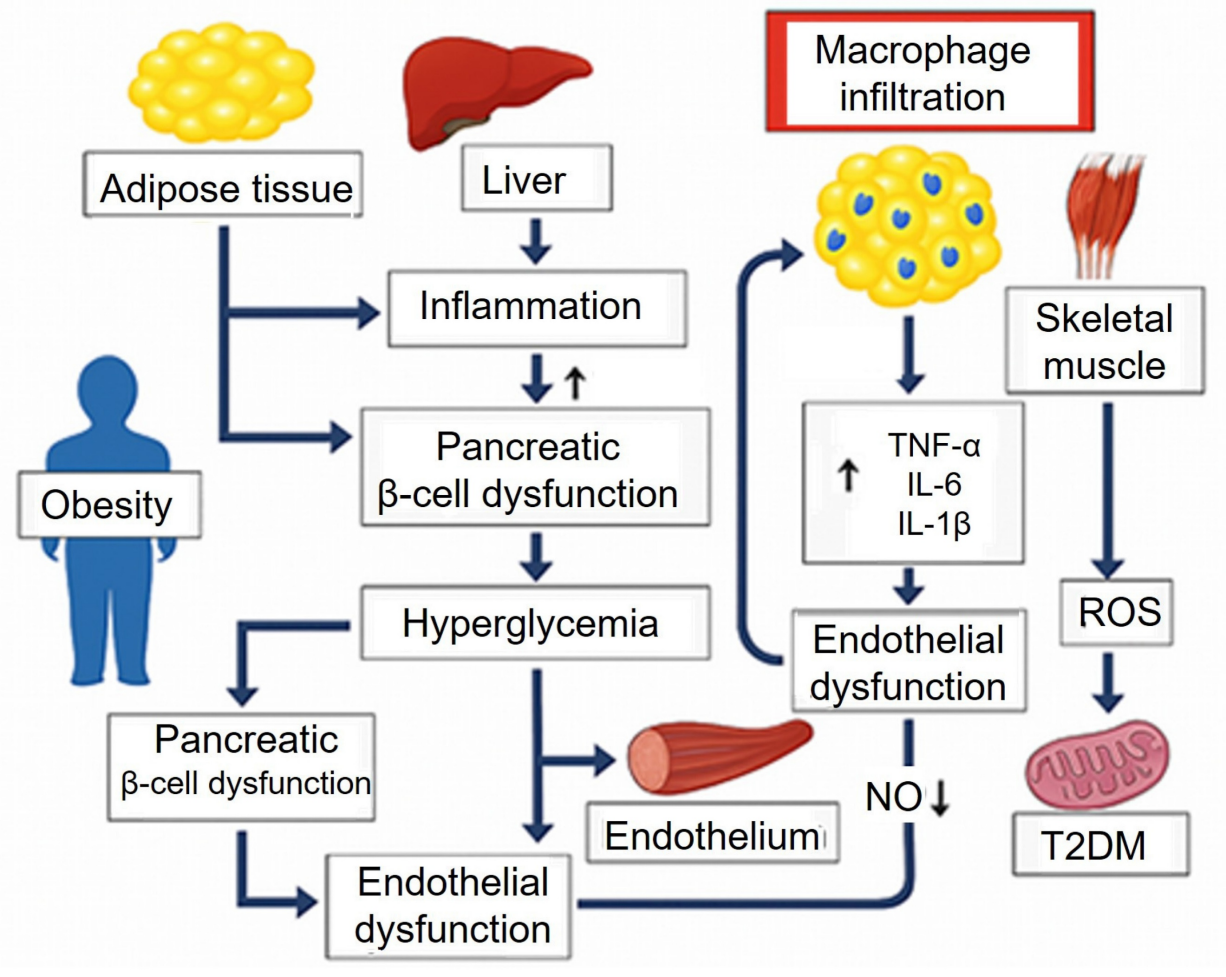
** Pathophysiological mechanisms linking insulin resistance, obesity, type 2 diabetes mellitus, and atherosclerosis.** Obesity and insulin resistance promote chronic inflammation, dyslipidemia, and endothelial dysfunction. These processes impair glucose metabolism, damage vascular integrity, and accelerate atherogenesis, contributing to the development and progression of type *2 diabetes mellitus* and atherosclerosis. AGEs: Advanced Glycation End-products; IL: interleukin; NF-kB: Nuclear Factor kappa-light-chain-enhancer of activated B cells; NO: Nitric Oxide; ROS: Reactive Oxygen Species; T2DM: Type 2 Diabetes Mellitus; TNF-α: Tumor Necrosis Factor-alpha.

*Diabetes mellitus* represents a complex interplay of metabolic and endocrine disturbances that often coexist and exacerbate other endocrine disorders. These conditions are interlinked through common mechanisms such as insulin resistance, dysregulation of hormonal axes, chronic inflammation, and metabolic disturbances, which not only affect endocrine function but also contribute to the development of atherosclerosis, impaired wound healing, and other vascular complications. The intricate relationships between these diseases (Figure 1) underscore the need for a unified approach to treatment that addresses the shared pathological mechanisms and their systemic effects.

One of the central features of type 2 *diabetes* mellitus (T2DM) is insulin resistance, where tissues such as skeletal muscle, liver, and adipose tissue become less responsive to insulin. This leads to elevated blood glucose levels (hyperglycemia) and dysregulated lipid metabolism, both of which contribute to the development of atherosclerosis, a process characterized by the thickening and hardening of arterial walls due to lipid deposition and inflammation 26. Insulin resistance also promotes endothelial dysfunction, an early event in atherosclerosis, where the ability of blood vessels to dilate and contract properly is impaired. This is compounded by increased levels of pro-inflammatory cytokines, such as tumor necrosis factor-alpha (TNF-α) and interleukin-6 (IL-6), which promote the recruitment of immune cells to the vessel walls and increase vascular permeability, further accelerating the atherosclerotic process 27.

Obesity, often a precursor to insulin resistance, exacerbates this process. Visceral fat in particular releases pro-inflammatory cytokines and adipokines, such as adiponectin and leptin, which influence insulin sensitivity and promote inflammatory pathways in the vasculature, accelerating the progression of atherosclerosis 28. Additionally, hyperglycemia in diabetes increases the accumulation of advanced glycation end-products, leading to further endothelial injury, oxidative stress, and inflammation.

Impaired wound healing is a hallmark of *diabetes mellitus*, particularly in patients with poorly controlled blood glucose levels. High glucose levels disrupt the normal function of keratinocytes and fibroblasts, impair collagen deposition, and reduce angiogenesis, all of which are essential for effective wound healing. Moreover, chronic inflammation and increased oxidative stress in diabetes exacerbate tissue damage and delay the repair process. In addition to diabetes, hypothyroidism contributes to poor wound healing by impairing collagen synthesis and reducing fibroblast activity 29.

The interlinked mechanisms reveal a shared pathophysiological foundation involving insulin resistance, chronic inflammation, endothelial dysfunction, and metabolic dysregulation. These conditions often coexist and exacerbate one another. Understanding both the shared and distinct pathways underlying these disorders enables the development of more precise and effective therapeutic strategies. The NZ approaches should not only target the specific endocrine dysfunctions but also address the systemic metabolic and vascular disruptions they provoke 30.

## 5. Nanozymes in *Diabetes Mellitus*: diagnostic, therapeutic, and nanotheranostic applications

### 5.1. Diagnostic applications of nanozymes

#### Glucose detection in blood, urine, and saliva. Portable and efficient devices

One of the primary diagnostic uses of NZs in diabetes management is the detection of glucose levels in biological fluids such as blood, urine, or saliva. For instance, Lee et al. developed a glucose oxidase-conjugated graphene oxide/MnO_2_ nanozyme, which exhibited horseradish peroxidase-mimic activity to catalyze the oxidation of 3,3′,5,5′-tetramethylbenzidine (TMB) in the presence of H_2_O_2_, allowing direct detection of glucose in whole blood. This platform demonstrated a wide linear quantification range from 25 mg/dL to 300 mg/dL, providing a method for glucose monitoring without the need for blood sample pretreatment 31. Similarly, Karim et al. developed a silver nanoparticle-embedded cotton fabric-based nanozyme sensor for the colorimetric detection of glucose in urine. This system was advantageous due to the high surface area of cotton fibers that facilitated the adsorption and detection of glucose molecules, allowing for rapid glucose measurements in urine 32. Also, Naveen Prasad et al. introduced a copper nanozyme for non-invasive glucose detection in human urine. The copper nanozyme demonstrated the ability to catalyze the oxidation of TMB to its double oxidation diimine product, rather than the charge-transfer complex, providing a glucose measurement with minimal sample processing 33. Roy et al. evaluated biocompatible nano-hybrids composed of Mn_3_O_4_ nanoparticles for the non-invasive detection of glucose in human saliva 34.

NZ-based colorimetric biosensors for noninvasive glucose monitoring have been the subject of extensive scientific investigation. As recently reviewed by Jeon et al. 35, other biofluids analyzed included sweat, tears, and interstitial fluids. The primary detection methods employed were optical and electrochemical techniques. Among the nanomaterials used, AuNZs were the most common, although a diverse range of other NZs have also been explored, including CuInS₂, SiO₂, ZnO, and CuO 35. Yi et al. reported a smartphone-based, paper-based analytical device utilizing an acetylene black-hemin NZ complex for glucose detection in blood. Compared with graphene oxide-supported hemin, acetylene black-hemin exhibited superior signal response on paper. The device provided a linear detection range from 3.6 mg/dL to 540 mg/dL, demonstrating potential for use in diabetes diagnostics 36. Similarly, Khachornsakkul et al. developed a microfluidic paper-based analytical device using gold and silver nanoparticles as peroxidase-like NZs and a colorimetric probe to monitor glucose levels using a smartphone application, achieving clinically relevant glucose detection in 20 minutes 37.

***Achievements and challenges*.** As shown, recent progress in NZs has greatly advanced colorimetric-based glucose biosensing. Their use enhances the practicality of simple, visual glucose detection, particularly when combined with digital tools like smartphone-based analysis. The reported studies, nevertheless, stand only at the *in vitro* diagnostic level, highlighting both the promise and the current limitations of this field. However, these cases demonstrate how NZs have been adapted to various biological fluids (blood, urine, saliva, sweat, and tears), each with tailored design strategies. For instance, graphene oxide/MnO₂ NZs enable direct blood glucose detection without pretreatment, while silver nanoparticle-embedded cotton fabrics enhance surface interaction for rapid urine analysis. Copper NZs improve signal clarity by producing stable diimine products, and Mn₃O₄ hybrids offer biocompatibility for saliva-based sensing. Smartphone-integrated platforms using acetylene black-hemin or gold/silver NZs in paper-based and microfluidic formats provide fast, portable, and user-friendly diagnostics. Despite these advances, several challenges remain. Most NZs primarily mimic peroxidase activity, limiting the range of catalytic functions and detectable analytes. The accuracy of colorimetric detection is also affected by environmental conditions and variations in smartphone camera performance, reducing reliability and reproducibility. Moreover, potential toxicity, especially from metal-based NZs, raises safety concerns for wearable or implantable applications, necessitating further toxicological studies. Machine learning offers promising solutions by enabling smarter nanozyme design and more accurate image analysis. Nonetheless, limitations in catalytic activity, fabrication complexity, and safety still hinder clinical and commercial adoption. Continued interdisciplinary research is essential to overcome these barriers and fully realize the potential of nanozyme-based biosensors.

### 5.2. Therapeutic and theragnostic potential of nanozymes in *Diabetes Mellitus*

The application of NZs in the treatment of *diabetes mellitus* is based on their ability to modulate oxidative stress, inflammation, insulin resistance, and hyperglycemia, which are critical contributors to the pathogenesis of diabetes.

#### Therapeutic applications improving insulin resistance

Wang et al. 38 demonstrated the therapeutic potential of Au@Pt NZs, which were found to enhance glucose tolerance and reduce triglyceride levels in high-fat diet-induced diabetic mice. Their multi-omics analysis revealed that Au@Pt NZs treatment modulated critical metabolic pathways such as glycolysis, pyruvate metabolism, PPAR signaling, and insulin signaling. The findings suggest that Au@Pt NZs can target both the liver and the gut microbiome 38. Similarly, Song et al. 39 explored the use of a single-atom Ce-N_4_-C-(OH)_2_ NZs that promoted glucose absorption in lysosomes, leading to increased reactive ROS production in human hepatic HepG2 cells and initiating a cascade reaction involving SOD-like, oxidase-like, catalase-like, and peroxidase-like activities. This significantly improved insulin sensitivity and glucose tolerance in diabetic mice without significant toxicity 39. In another study, Zhou et al. 40 used Fe_3_O_4_ NZs to activate adenosine monophosphate-activated protein kinase (AMPK), an essential regulator of glucose metabolism, within lysosomes. By enhancing glucose uptake and insulin sensitivity, Fe_3_O_4_ NZs improved the metabolic profile in genetically and diet-induced diabetic models. This emphasizes the organelle-specific properties of Fe_3_O_4_ NZs in managing hyperglycemia and insulin resistance 40. Lin et al. 41 introduced a single-atom Ce-N-C NZ that improved insulin resistance and promoted glucose metabolism in both cell cultures and mouse models of type 2 diabetes. All these studies were conducted at the *in vivo* level in diabetic mice but have not yet progressed to human clinical studies.

#### Therapeutic applications targeting oxidative stress

Zhang et al. 42 targeted the liver using a biodegradable silica nanoshell embedded with platinum NZs (Pt-SiO_2_) acting as a ROS scavenger, mitigating oxidative stress and restoring glucose consumption in the liver. The Pt-SiO_2_ NZ significantly improved glucose tolerance, insulin resistance, and lipid accumulation in diabetic mice. Moreover, Li et al. 43 demonstrated the efficacy of CeO_2_ NZs in improving skeletal muscle function in gestational *diabetes mellitus* offspring. By attenuating mitochondrial oxidative stress and restoring mitochondrial activity, CeO_2_ NZs enhanced insulin sensitivity and skeletal muscle motility, suggesting a potential therapeutic approach for mitigating the long-term effects of gestational diabetes on offspring.

#### Therapeutic applications modulating inflammation

Shen et al. 44 designed AuCePt porous hollow cascade NZs for targeted delivery of disulfiram, a drug known to reduce hepatic insulin resistance. The multifunctional AuCePt NZs exhibited SOD and catalase-like activities, enabling them to scavenge ROS and improve glucose uptake in insulin-resistant hepatocytes. The targeted drug delivery system demonstrated therapeutic effects in diabetic mice 44.

#### Therapeutic applications regulating hyperglycemia and glucose homeostasis

Therapeutic strategies may additionally focus on direct regulation of glucose levels. For example, Ce-N₄-C NZs improved glucose tolerance through lysosomal modulation 39. Kim and Kim 45 developed a glucose-responsive protein delivery system model enabling glucose-responsive protein release *in vitro*, offering potential for closed-loop insulin delivery in diabetes management.

#### Theranostic approaches

**Figure 3:**
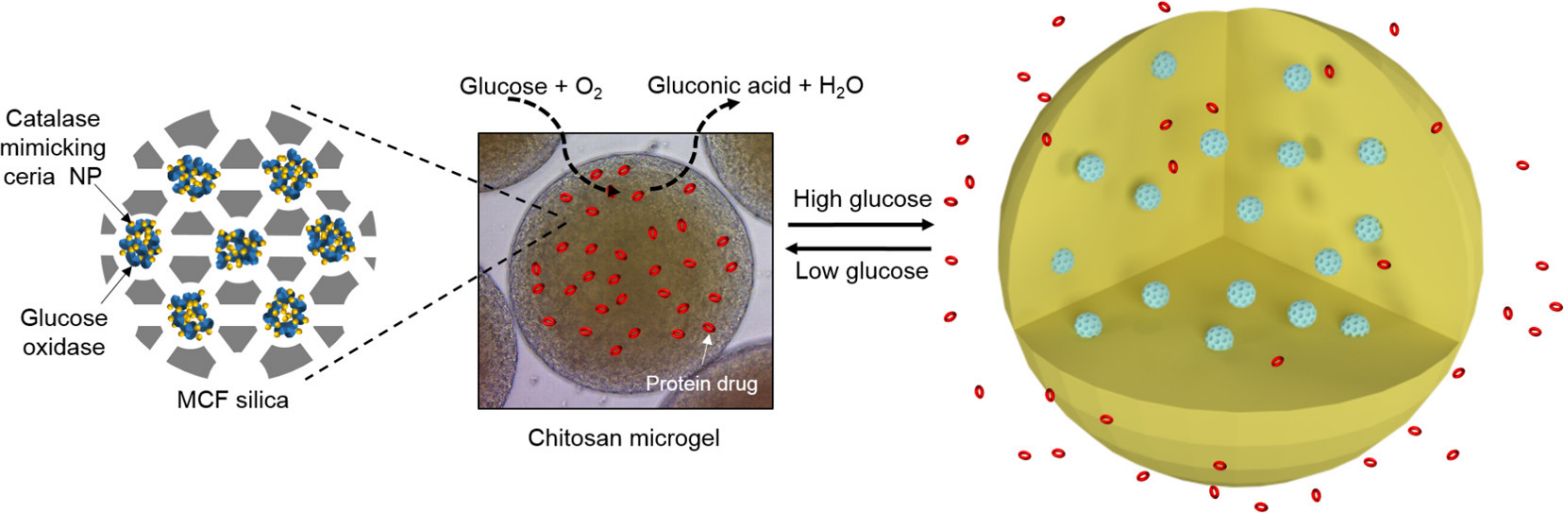
** Glucose-responsive chitosan microgels for self-regulated insulin delivery.** A glucose-responsive insulin delivery system was developed using pH-sensitive chitosan microgels loaded with mesoporous silica and ceria nanoparticles. Under hyperglycemic conditions, enzymatic reactions lower the pH, causing microgel swelling and controlled insulin release, with ceria nanoparticles protecting the enzymatic activity by decomposing H_2_O_2_. Reprinted with permission from Kim et al. 45. Copyright © 2017, American Chemical Society.Final del formulario

**Figure 4:**
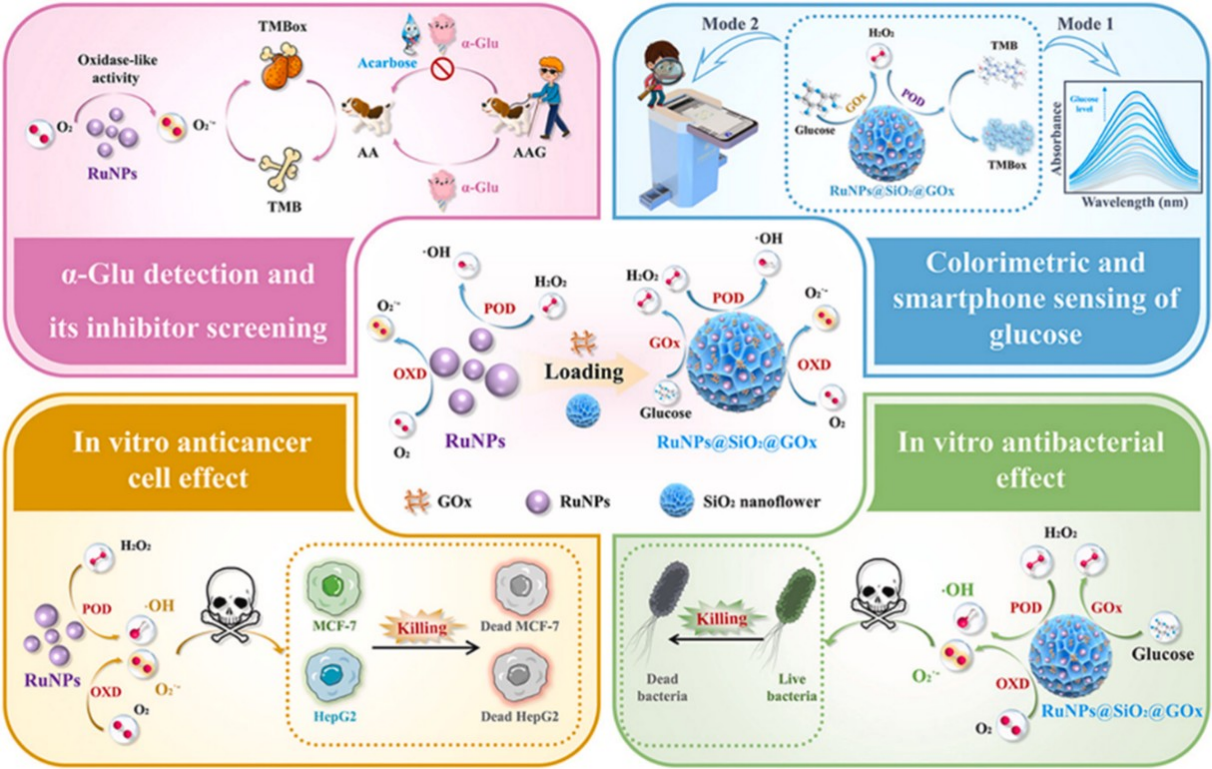
** Multifunctional applications of Ruthenium nanozymes (RuNz).** RuNZs were used for disease marker detection, cancer cell elimination, and antibacterial applications. A colorimetric biosensor for α-glucosidase (GOx) activity was designed. Additionally, a RuNPs@SiO2@GOx-based nanoreactor was developed for blood glucose detection using a smartphone-integrated platform. The multifunctional RuNZs also showed potential in anticancer and antibacterial applications. Reprinted with permission from Wang et al. 46. Copyright © 2024, Elsevier B.V.

In addition to therapeutic applications described above, NZs have gained significant attention for their theragnostic potential, combining diagnostic and therapeutic functions in a single platform. As previously mentioned, Kim and Kim 45 developed a glucose-responsive protein delivery system utilizing chitosan microgels integrated with enzyme-mimicking inorganic nanoparticles. These pH-sensitive microgels were fabricated via electrospray and embedded with mesocellular foam (MCF) of large-pore mesoporous silica. The MCF was loaded with CeO_2_ NZs, which exhibit catalase-like activity, and glucose oxidase (GOx). In hyperglycemic conditions, GOx catalyzes glucose oxidation, producing H₂O₂. CeO_2_ decomposes the H₂O₂, regenerating oxygen and protecting GOx from denaturation. This enzymatic activity lowers the local pH, causing the chitosan microgels to swell and release encapsulated proteins, such as bovine serum albumin and insulin. The system demonstrated basal release under normoglycemic conditions and enhanced release under hyperglycemic conditions, offering potential for closed-loop insulin delivery in diabetes management (Figure 3). In a recent study, Wang et al. 46 synthesized ruthenium NZ (RuNZs) with both oxidase and peroxidase activities through a solvothermal method. These RuNZs were employed in a multifunctional nanoplatform for *in vitro* disease marker detection, cancer cell elimination, and antibacterial applications. Leveraging their oxidase (GOx) activity, the researchers developed a colorimetric biosensor to measure α-glucosidase activity and screen potential inhibitors. Additionally, a RuNZs@SiO₂@GOx nanoreactor was engineered, integrating biological enzymes with NZs, to create a colorimetric sensing platform for rapid blood glucose detection. The multifunctional RuNZs also demonstrated efficacy in anticancer and antibacterial activities, broadening the potential applications of NZs in theranostics (Figure 4).

**Figure 5:**
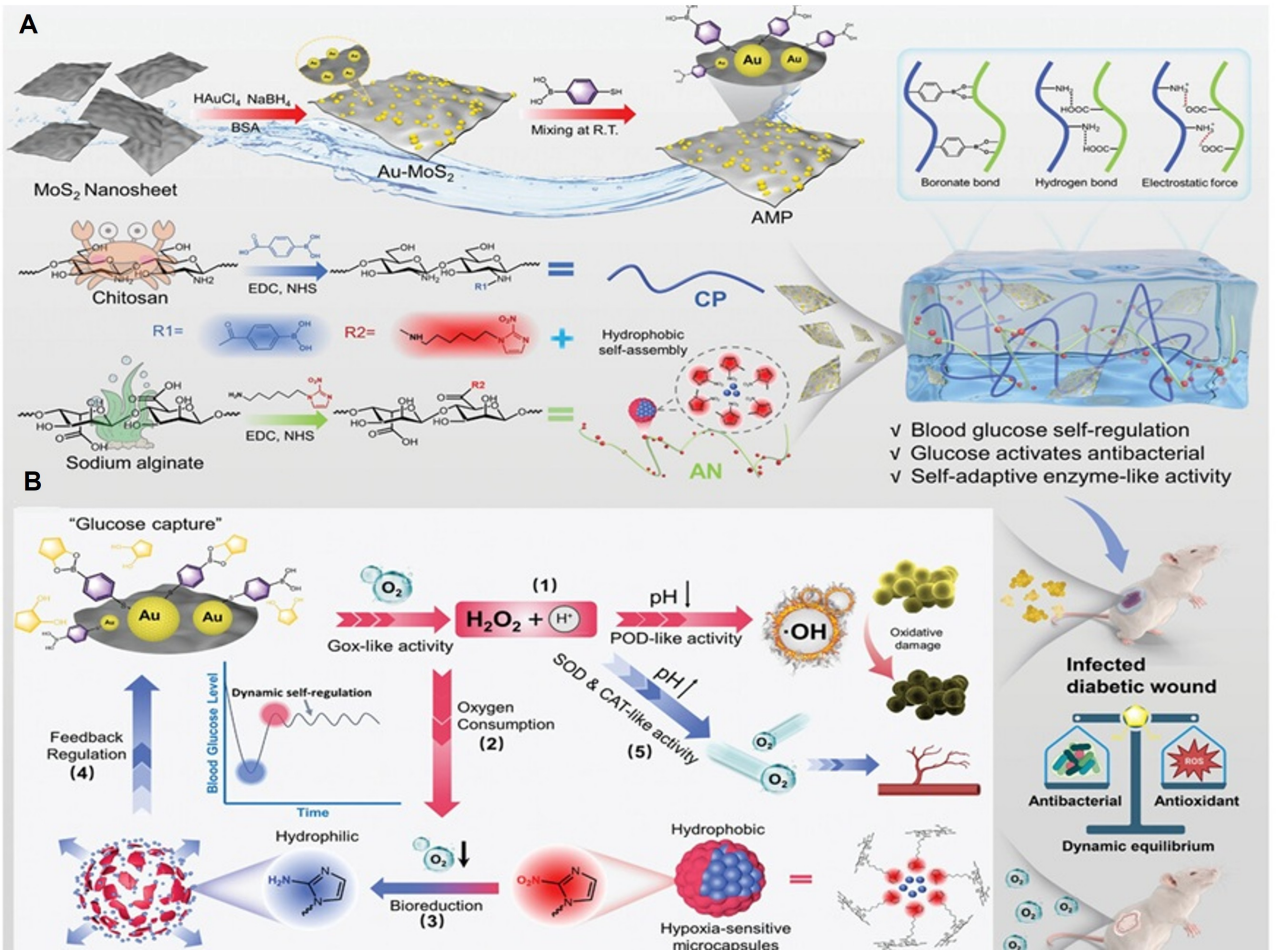
Construction (A) and tissue regeneration-promoting effects (B) of a glucose-activated programmed hydrogel for infected diabetic wound healing. The hydrogel combines phenylboronic-acid-modified chitosan, nitroimidazole-modified alginate, and Au-MoS₂ nanozymes. Under hyperglycemia, it generates ROS to kill bacteria and triggers insulin release in hypoxic conditions. In normoglycemia, it releases oxygen to relieve hypoxia. This example of a dynamic system enables feedback-controlled glucose regulation and promotes orderly wound healing. Reprinted with permission from Yang et al. 47. Copyright © 2025, Wiley.

Yang et al. 47 used a glucose-activated self-switching enzyme-like activity to program a hydrogel for regulating insulin release in response to blood glucose fluctuations, allowing for both blood glucose management and wound healing. The hydrogel was composed of Au and MoS_2_ NZs, and insulin was loaded into hypoxia-sensitive microcapsules. In hyperglycemia, glucose generated ROS for antibacterial activity and triggered insulin for blood glucose regulation. In a normoglycemia environment, the enzyme-like activity supplied oxygen, inhibiting further insulin release and facilitating wound recovery in mice through blood glucose regulation and an improved wound microenvironment (Figure 5).

#### Nanozymes in diabetic wound healing

Among the different therapeutic aspects of diabetes, the field of diabetic wound healing, particularly concerning diabetic foot ulcers (DFUs), has garnered significant attention and scientific output. A recent review has compiled and analyzed key studies related to DFUs, highlighting the prominence of this area in diabetes research 48. Therefore, we include here only recent works focusing on diabetic wound healing.​ DFUs are chronic, non-healing wounds resulting from diabetic neuropathy, vascular disease, and bacterial infections. The complexity of DFUs arises from a multifaceted microenvironment characterized by recurrent infections, excessive oxidative stress, persistent inflammation, and ischemia-hypoxia, all secondary to hyperglycemia 49. Current clinical treatments often yield suboptimal outcomes, and the development of new treatments is a priority to improve the quality of life for diabetic patients 50. Compared to natural enzymes, NZs offer more stable catalytic activity, lower production costs, and greater maneuverability. Notably, many NZ exhibit multienzyme activities, enabling them to catalyze multiple enzyme-like reactions simultaneously throughout the DFU recovery process. Additionally, their favorable photothermal and acoustic properties can be exploited to further enhance therapeutic effects.

The recent studies on NZs for wound healing are summarized in Table 2. NZs have been integrated into various hydrogel and microneedle systems to enhance wound healing through multiple mechanisms. These include scavenging of ROS, generating oxygen, modulating immune responses, and providing antibacterial effects. NZs are often combined with other therapeutic agents, such as growth factors and antimicrobial peptides, to create multifunctional wound dressings that address the complex microenvironment of diabetic wounds. The studies demonstrate that NZ-based treatments can accelerate wound closure, reduce inflammation, promote angiogenesis, and improve collagen deposition, leading to enhanced healing outcomes. For example, Wei et al. 51 reported a selenium-based Janus liposozyme that promoted macrophage polarization and tissue regeneration in a large-animal model (mini pigs), providing rare evidence of NZs efficacy beyond rodent studies. Similarly, MnO₂-loaded hydrogels 52 not only scavenged ROS and generated oxygen and nitric oxide but also reduced neutrophil infiltration, modulated cytokine expression, and promoted neovascularization, illustrating the multi-targeted potential of NZ therapies in diabetic wounds.

The scientific literature reveals that different targeting strategies have been employed in NZ-based therapies to promote diabetic wound healing, including modulation of oxidative stress, inflammation, angiogenesis, and antibacterial activity. Targeting oxidative stress has been a major approach in NZ-based wound healing. Several NZ-based hydrogels or composite systems scavenge ROS and generate oxygen to restore redox balance in DFUs. Examples include Pt-SiO₂, CeO₂, and MnO₂ NZ, which demonstrated reduced oxidative injury, improved mitochondrial activity, and enhanced angiogenesis 60,67,68,75,89. Additional systems such as Cu-based NZs assembled with polyphenol ligands 80 and Zn-based polymetallic constructs 78 further contributed to ROS scavenging and collagen regeneration. These systems collectively reduce oxidative injury, restore cellular metabolism, and accelerate wound closure. All of these studies have been conducted *in vitro* and in animal models, primarily in diabetic rats and mice (Table 2), except the study by Wei et al. 51, which also employed mini pigs. To date, no clinical studies have been reported.

Inflammation modulation represents another critical strategy. NZ therapies attenuate inflammatory cascades by lowering pro-inflammatory cytokines (e.g., IL-1β, IL-6, TNF-α) and promoting macrophage polarization toward a regenerative phenotype. Representative systems include MnO₂-based hydrogels and Cu/Zr MOFs 52,53,63,72. PtCuTe nanosheets 65, Pt hydrogels 66, and CuCeO₂ NZs 85 also demonstrated NF-κB inhibition and Nrf2 activation, enhancing granulation tissue formation and immune homeostasis**.** These anti-inflammatory effects are crucial for transitioning wounds from the inflammatory to the proliferative phase.

Promotion of angiogenesis and tissue regeneration is also essential in DFU recovery. Certain NZs directly stimulate angiogenesis and collagen deposition, thereby accelerating wound closure. Examples include Prussian blue NZ microneedles with VEGF loading, FeS/Au hybrids upregulating HIF-1 and VEGF, and Zn-based polymetallic constructs that enhanced collagen regeneration 60,73,78. Other systems, such as Cu-ZnO hydrogels 76, Pt-based hydrogels 61,99, and Au-Pt NZs 83 supported endothelial cell function and osteogenic differentiation.

Finally, antibacterial strategies play a key role in addressing DFUs, which are prone to recurrent infections. NZs such as Au/MoS₂, Au-Pt hydrogels, and aptamer-functionalized glucose oxidase NZ demonstrated strong antibacterial efficacy in wound models, either through ROS generation or pathogen-triggered activation 54,56,58. Additional antibacterial systems include Fe₂(MoO₄)₃ NZs 59, Cu-MOFs 102, and FeS aerogels 103, which were effective against biofilms and resistant pathogens.

## 6. Potential role of nanozymes in atherosclerosis and obesity

### 6.1. Interlinking metabolic disorders: diabetes, atherosclerosis, and obesity

The global surge in metabolic disorders has underscored the critical interrelationship between *diabetes mellitus*, atherosclerosis, and obesity, often referred to as the metabolic triad. Obesity is a major risk factor for both T2DM and atherosclerosis due to its strong association with chronic low-grade inflammation, insulin resistance, and dyslipidemia. Excess adipose tissue secretes pro-inflammatory adipokines that not only impair glucose metabolism but also contribute to endothelial dysfunction and oxidative stress, setting the stage for vascular complications 104. T2DM exacerbates atherosclerotic processes by promoting glycation of lipoproteins, enhancing oxidative stress, and activating inflammatory pathways that destabilize atherosclerotic plaques. Hyperglycemia further induces endothelial damage, accelerates foam cell formation, and enhances monocyte adhesion. Meanwhile, atherosclerosis can impair insulin delivery and exacerbate diabetic complications by reducing vascular perfusion 105. Given their overlapping pathophysiological features, therapeutic strategies targeting these shared mechanisms hold promise for simultaneously addressing obesity, T2DM, and atherosclerosis. In this context, NZs have emerged as multifunctional platforms capable of modulating oxidative and inflammatory pathways, offering novel therapeutic opportunities across these interconnected conditions.

### 6.2. Nanozymes in atherosclerosis therapy

Atherosclerosis, a chronic inflammatory disease characterized by plaque formation and lipid accumulation in arterial walls, has shown promising therapeutic responses to nanozyme-based interventions. NZs can address several pathological hallmarks of atherosclerosis, including oxidative stress, inflammation, and foam cell formation. Chen et al. 106 designed a biomimetic nanozyme platform by coating cerium-doped supra-carbon dots with macrophage membranes to achieve ROS-responsive theragnostic for atherosclerosis. These nanoparticles demonstrated dual-mode imaging capabilities (fluorescence and photoacoustic) and cascade enzymatic activity activated by the plaque's oxidative microenvironment. Functionally, the NZ platform enabled precise imaging and reduced ROS levels, suppressed M1 macrophage infiltration, and inhibited foam cell formation, effectively regulating the plaque microenvironment and slowing atherogenesis.

Another multifunctional approach involved Prussian blue-based NZs encapsulated with bovine serum albumin and curcumin. Xu et al. 107 demonstrated ROS scavenging and reduced pro-inflammatory cytokines such as TNF-α and IL-1β in foam cells. Additionally, it promoted cholesterol efflux by upregulating ABCA1 (ATP-binding cassette transporter A1) and ABCG1 (ATP-binding cassette transporter G1) in foam cells, ultimately decreasing lipid burden and metalloproteinase activity within plaques.

Expanding on this, Wang et al. 108 developed CS-Lip/PB@Rap, a chondroitin sulfate-modified Prussian blue nanozyme loaded with rapamycin. This system exhibited dual-targeting capabilities via CD44 receptors on inflammatory macrophages and vascular smooth muscle cells. Its enzyme-like properties allowed it to restore redox homeostasis and disrupt inflammatory crosstalk by suppressing NF-κB-mediated cytokine feedback loops. The nanozyme also attenuated vascular smooth muscle cells' phenotype switching and ox-LDL formation, both crucial in plaque destabilization.

Chen et al. 109 further contributed to the field with CLIKKPF-peptide-functionalized carbon-dot NZs, which provided real-time monitoring of plaque progression and showed significant ROS-scavenging and anti-inflammatory effects. Their specific binding to phosphatidylserine on foam cell membranes enhanced selective plaque targeting, optimizing both diagnostic precision and therapeutic outcomes.

#### Key takeaways

- Nanozyme types: CeO₂, Prussian blue, and manganese-based.
- Mechanisms: Mimic antioxidant enzymes (SOD, CAT, glutathione peroxidase) to reduce ROS, stabilize plaques, and regulate macrophage polarization.
- Translational status: Preclinical models show strong potential for anti-inflammatory and plaque-stabilizing therapies; still in early-stage development.

### 6.3. Nanozymes in obesity and metabolic dysfunction

As a multifactorial metabolic disorder characterized by chronic inflammation, oxidative stress, and lipid dysregulation, obesity presents complex therapeutic challenges. Antioxidant and immunomodulatory properties of NZs offer a novel paradigm for addressing obesity-related comorbidities.

Parra-Robert et al. 110 developed mesoporous silica-coated cerium oxide (CeO₂) NZs that significantly improved lipid profiles and reduced systemic inflammation in obese Zucker rats. These NZs modulated hepatic and adipose gene expression and downregulated the PI3K/mTOR/AKT signaling pathway, contributing to long-term metabolic improvements.

In a combined mouse model of non-alcoholic steatohepatitis and alcohol-associated liver disease, Gopal et al. 111 reported that nanoformulated SOD1 ameliorated liver damage and systemic inflammation. Notably, the NZs reduced CYP2E1 (Cytochrome P450 2E1) expression in visceral adipose tissue and increased SOD1 activity in the liver, curbing oxidative damage and metabolic stress.

Macrophage polarization plays a pivotal role in obesity-related inflammation. Duan et al. 112 identified CD146 as a key regulator of pro-inflammatory macrophage activation in obese adipose tissue. Targeting the CD146/Gp130 complex with antibodies in obese mice modulated JNK and STAT3 signaling pathways, shifting macrophage polarization toward an anti-inflammatory phenotype and improving insulin sensitivity.

Ding et al. 113 introduced aptamer-modified gold nanoclusters as targeted NZs for ROS scavenging in white adipocytes *in vitro*. These nanoclusters exhibited high SOD- and catalase-mimetic activity and strong adipocyte-specific targeting, effectively reducing oxidative stress with minimal toxicity, offering a tailored approach for managing obesity-induced oxidative burden.

Lastly, Natarajan et al. 114 demonstrated that copper/zinc SOD NZs protected against ethanol-induced hepatic steatosis and adipose inflammation in mice. The NZ therapy modulated lipogenic transcription factors such as SREBP-1c and enhanced AMPK phosphorylation, while increasing anti-inflammatory markers in adipose tissue, showcasing their dual-organ therapeutic potential.

#### Key takeaways

- Nanozyme types: CeO₂, iron oxide, and carbon-based.
- Mechanisms: Regulate oxidative stress, modulate mitochondrial function, and improve insulin sensitivity.
- Translational status: mostly animal model studies; translational pipeline still emerging.

### 6.4. Achievements and challenges

As shown, a transformative class of therapeutic agents capable of mimicking natural enzymatic activities with enhanced stability, tunability, and multifunctionality has been satisfactorily evaluated in different models of disease. Their application in addressing chronic metabolic alterations in atherosclerosis and obesity has shown substantial promise, particularly through their ability to modulate oxidative stress, regulate inflammatory pathways, and target pathological microenvironments with high specificity. Globally, for atherosclerosis, NZs, such as Prussian blue derivatives, CeO_2_-based nanostructures, and functionalized carbon dots have demonstrated robust ROS-scavenging and anti-inflammatory properties, enabling suppression of foam cell formation, stabilization of plaques, and even real-time imaging of lesion sites through integrated theragnostic modalities. Similarly, in the context of obesity, NZs, like CeO₂, nanoformulated SOD1, and aptamer-conjugated gold nanoclusters have effectively improved adipose tissue oxidative status, reduced pro-inflammatory macrophage infiltration, and ameliorated hepatic and systemic metabolic dysregulation.

## 7. Potential role of nanozymes in other endocrine disorders

The application of NZs in biomedical diagnostics and therapeutics has been comparatively underexplored in endocrine disorders beyond diabetes and obesity. The primary advantages explored thus far include their seamless integration into point-of-care diagnostic platforms. In recent years, NZs have demonstrated significant potential across a spectrum of endocrine disorders, particularly in enhancing the sensitivity and specificity of biomarker detection. Nevertheless, early studies reveal considerable potential, especially in terms of diagnostic precision, therapeutic targeting, and integration into point-of-care platforms.

### 7.1. Nanozymes in Cushing's syndrome and autonomous hypercortisolism

Adrenal hyperfunction, particularly in Cushing's syndrome, whether adrenocorticotropic hormone (ACTH, a pituitary hormone that stimulates cortisol secretion) dependent or independent, may affect glucose metabolism and induce cardiovascular diseases. Specifically, Cushing's syndrome and the less severe autonomous cortisol secretion phenotype are characterized by central obesity, hypertension, insulin resistance, hyperglycemia, and an altered coagulability, all of which accelerate the development of atherosclerosis 115. Increased deposition of visceral fat worsens the inflammatory milieu in the vasculature, insulin resistance, and endothelial dysfunction 116,117. Moreover, Cushing's syndrome interferes with wound healing through the excess of cortisol that suppresses immune function, reduces fibroblast proliferation, and delays the inflammatory phase of wound healing. The associated chronic tissue inflammation and hyperglycemia may also inhibit tissue regeneration mechanisms 115. Addison's disease (hypocortisolism), on the other hand, results in adrenal insufficiency, leading to metabolic disturbances, including hypoglycemia and electrolyte imbalance 118. Diagnosis of these entities poses diagnostic challenges due to fluctuating hormone levels and the limited sensitivity of conventional assays. Recent studies have demonstrated how NZs can transform the detection landscape for these conditions. Du et al. 119 developed an ultrasensitive electrochemical immunosensor for cortisol detection using gold single-atom NZs. These NZs, in combination with horseradish peroxidase-labeled antibodies, achieved a highly sensitive detection platform through an Au-S bond-mediated conjugation, offering improved detection limits and signal amplification in hormone assays. Similarly, Yang et al. used a label-free electrochemical immunosensor with a metal-organic framework (SnS_2_/NiCo MOFs) for the sensitive detection of cortisol 120. Silver nanoclusters have also been used to achieve low limits of detection in cortisol measurements 121.

#### Key takeaways

- Nanozyme types: Au, MOF-based NZ (SnS₂/NiCo), and Ag nanoclusters.
- Mechanisms: Enhanced electrochemical and photoelectrochemical signal amplification for cortisol detection, enabling highly sensitive and rapid assays.
- Translational status: Proof-of-concept diagnostic studies only; no therapeutic applications yet, but strong potential for point-of-care testing in endocrine practice.

### 7.2. Nanozymes in thyroid disorders

Thyroid diseases may have some metabolic effects that overlap with diabetes and obesity. In hypothyroidism, there is a reduction in basal metabolic rate, leading to weight gain and an increase in serum cholesterol levels, both of which contribute to atherosclerotic cardiovascular disease. On the other hand, hyperthyroidism results in increased metabolism, but this can lead to increased cardiac output, arrhythmias, and, paradoxically, cardiovascular issues such as left ventricular hypertrophy 122. Furthermore, thyroid hormones play an important role in regulating lipid metabolism, and thyroid dysfunction can exacerbate the lipid disturbances seen in both diabetes and obesity, further increasing the risk of atherosclerosis and cardiovascular morbidity.

One promising strategy involves the development of NZ-based colorimetric sensors for the detection of antithyroid drugs. Zhang et al. 123 designed a system based on iron single atoms encapsulated in nitrogen and phosphorus co-doped carbon nanosheets, which exhibited enhanced peroxidase-like activity. This catalytic activity enabled the oxidation of TMB into a visually detectable blue product (oxTMB). Notably, methimazole, a commonly used antithyroid drug, acted as a peroxidase inhibitor in this system, effectively reducing oxTMB and causing the blue color to fade. Leveraging this mechanism, the authors established a visual, dual-functional sensor capable of detecting both methimazole and H_2_O_2_ with linear ranges of 5-50 mM and 3-50 mM, respectively, and a limit of detection for methimazole of 1.57 mM. This approach underscores the potential of NZs for rapid, accessible diagnostics in thyroid disorders.

In an elegant study, Wu et al. 124 explored the role of the NZ coordination environment in mimicking the natural inhibition patterns of thyroid peroxidase, the enzyme targeted by antithyroid drugs. They developed NZs with atomically dispersed CuN₄ catalytic sites, modified with adjacent hydroxyl groups (CuNC-OH), which mimicked the enzymatic pocket of thyroid peroxidase. This biomimetic configuration allowed for highly specific interactions with antithyroid drugs, offering a new platform for the high-throughput screening of candidate compounds. Importantly, this system provided a significant cost advantage over traditional enzyme-based kits, positioning nanozyme-assisted drug discovery as a scalable alternative in endocrine pharmacology.

Moving from diagnostics to therapy, Wang et al. 125 introduced a multifunctional nanozyme-based platform for the treatment of thyroid cancer. Their design incorporated platinum nanoparticles within hollow polydopamine carriers, which were loaded with the sonosensitizer Chlorin e6 and the chemotherapeutic agent lenvatinib. This nanoplatform was engineered to target galectin-3 receptors, which are overexpressed in thyroid cancer cells, facilitating receptor-mediated endocytosis. Upon ultrasound irradiation, the Chlorin e6 generated ROS, inducing oxidative stress and apoptosis. Simultaneously, the embedded platinum nanoparticles catalyzed the decomposition of H_2_O_2_ into oxygen, thereby alleviating tumor hypoxia and enhancing the therapeutic effect. Transcriptomic analyses further confirmed the efficacy of the treatment in promoting cancer cell apoptosis. This approach illustrates the therapeutic versatility of NZs when combined with targeted delivery and multimodal treatment strategies.

#### Key takeaways

- Nanozyme types: Au and CeO_2_.
- Mechanisms: Catalytic antioxidant activity reduces oxidative thyroid damage and supports hormone balance.
- Translational status: Proof-of-concept studies in thyroiditis and related dysfunctions; no clinical trials yet.

### 7.3. Nanozymes in chromaffin-secreting tumors

Chromaffin tumors, including pheochromocytoma and paragangliomas, are rare neuroendocrine neoplasms that arise from chromaffin cells of the adrenal medulla or extra-adrenal paraganglia. These tumors are characterized by excessive secretion of catecholamines, primarily epinephrine and norepinephrine, which leads to episodic hypertension, tachycardia, headaches, and metabolic disturbances. Their pathobiology involves genetic mutations (e.g., SDHx, RET, VHL) that affect mitochondrial function, redox balance, and cellular proliferation. The fluctuating nature of hormone secretion and the low abundance of circulating tumor cells (CTCs) pose diagnostic challenges due to fluctuating hormone levels and the limited sensitivity of conventional assays. NZs, with their enzyme-mimetic activity and signal amplification capabilities, offer promising solutions for improving the detection and monitoring of these tumors. Recently, Liu et al. 126 engineered a cascaded NZ-based microfluidic platform that integrates Au@CuMOF NZs with glucose meters and smartphone applications for the rapid detection CTCs associated with pheochromocytoma. This dual-enzyme mimetic system capitalized on glucose oxidase- and peroxidase-like activity to enable dual-mode (colorimetric and portable glucose meter-based) detection.

Several groups have harnessed laccase-like activity to detect catecholamines such as epinephrine and norepinephrine. Zhu et al. 127 synthesized nitrogen-doped carbon Co/CoOx NZs with strong laccase mimetic function, offering a simple and effective colorimetric platform for epinephrine detection. The system demonstrated a linear detection range suited for clinical diagnosis. Similarly, Kulandaivel et al. 128 designed a bioinspired copper-based coordination polymer capable of detecting both epinephrine and norepinephrine in complex biological matrices such as artificial urine, providing a strategy for monitoring treatment response in pheochromocytoma. The electrochemical and visual detection of pheochromocytoma-related CTCs has also seen advancement through a dual-mode platform developed by Liu et al. 129. This approach utilized a covalent organic framework-supported platinum NZ to amplify signals and enable highly sensitive cell detection, demonstrating the diagnostic relevance of NZs in low-abundance biomarker analysis. Huang et al. 130 further developed a layered double hydroxide-based NZ mimicking laccase activity for chromogenic detection of catecholamine biomarkers. This system, which used CuCoFe-based LDHzymes, allowed smartphone-assisted detection, reinforcing the suitability of NZs for portable diagnostics.

#### Key takeaways

- Nanozyme types: Iron oxide and hybrid nanozymes.
- Mechanisms: Exploit ROS generation and redox modulation for selective tumor suppression.
- Translational status: Limited to preliminary experimental data; requires further validation before clinical use.

### 7.4. Nanozymes in other endocrine-related disorders

***Reproductive axis.*** NZs have also been utilized to improve the detection of other key hormones, most notably human chorionic gonadotropin (hCG). Palladium-, platinum-, and ruthenium-based NZ have demonstrated excellent peroxidase-like activity. Wang et al. synthesized Pd@Pt-Ru dendritic NZs for ultrasensitive lateral flow immunoassays, enabling a 250-fold improvement in hCG detection sensitivity over conventional gold nanoparticles 131. Similarly, Cao et al. 132 introduced a “popcorn-like” Pd@Pt nanozyme, produced via green synthesis using *Cornus officinalis* extracts. This construct also significantly lowered the hCG detection threshold in lateral flow immunoassays. Yang et al. 133,134 reported the development of immunochromatographic test strips incorporating Au/Fe3O_4_ NZ and β-FeOOH nanorods. These platforms exhibited enhanced catalytic activity and improved signal amplification for hCG detection, with detection limits far surpassing traditional methods. Expanding beyond diagnostics, NZs are also being explored as therapeutic tools. Sun et al. 135 introduced a macrophage membrane-coated MnO_2_ nanosheet with nanozyme-like estrogen scavenging capabilities for the treatment of endometriosis. This therapeutic NZs inhibited estradiol-driven proliferation and inflammation *in vitro* and *in vivo*, presenting a promising nanomedical approach for hormone-dependent endometriosis.

**Circadian rhythm.** As mentioned, NZs have been employed in the detection of cortisol, the major potential source of circadian rhythm disruption. Interestingly, for evaluating the circadian rhythm, Wang et al. 136 developed a fluorescence-based sensor for melatonin quantification by integrating carbon dots with platinum/ruthenium NZs. The system enabled smartphone-compatible ratiometric sensing, with high sensitivity across physiological melatonin concentrations, highlighting the role of NZs in sleep and endocrine rhythm research.

**Gastrointestinal disorders.** In the gastrointestinal domain, Zheng et al. 137 proposed a dual-mode magnetic lateral flow assay using Fe_3_O_4_@Pt NZ for the detection of gastrin-17. The platform exhibited good sensitivity and specificity within clinically relevant ranges, enabling rapid, quantitative hormone testing suitable for conditions such as gastritis and gastric cancer screening.

### 7.5. Achievements and challenges

NZs have demonstrated considerable promise in the detection of hormones beyond those involved in metabolic regulation, such as hCG, melatonin, and gastrin, which are essential for reproductive and gastrointestinal health. The key advantages of using NZs in these contexts include their high catalytic efficiency, robustness under diverse environmental conditions, and compatibility with point-of-care diagnostic formats, allowing for rapid, low-cost, and potentially field-deployable hormone assays. Furthermore, their tunable surface properties enable selective detection, while their enzyme-mimetic activity supports signal amplification, enhancing sensitivity in low-abundance hormone detection. However, despite these benefits, the application of NZs in this area remains limited. The number of studies focusing on NZ-based detection of these specific hormones is still relatively small, which restricts the generalizability of current findings and the development of standardized protocols. Additionally, challenges remain regarding the specificity of NZ-hormone interactions in complex biological matrices, as well as the potential for interference by structurally similar molecules 138,139. Continued research and validation in clinical settings are necessary to fully realize the potential of NZs in hormone diagnostics and endocrine therapy.

## 8. Mechanistic insights, comparative benefits, translational gaps, and regulatory considerations

Schema 1 and Table 4 present an overview of NZ classes, highlighting their catalytic mechanisms, target organs, and applications in the treatment of endocrine and metabolic disorders. Catalytic activities directly influence biological processes central to endocrine disorders. These catalytic mechanisms are not merely biochemical reactions but are tightly coupled with disease-modifying pathways, offering a mechanistic rationale for the therapeutic and diagnostic use of NZs in endocrinology. Their SOD-like activity, for instance, mitigates oxidative stress in insulin-sensitive tissues such as the liver and skeletal muscle, thereby restoring mitochondrial function and improving insulin signaling. This mechanism is particularly relevant in type 2 diabetes and obesity, where chronic ROS accumulation impairs glucose uptake and lipid metabolism. Catalase-mimetic nanozymes further detoxify H_2_O_2_, promoting tissue regeneration and angiogenesis, which are key processes in diabetic wound healing and vascular repair. Peroxidase-like NZs, such as those based on iron oxide or Prussian blue, modulate inflammatory cascades by reducing NF-κB activation and cytokine release, which is critical in atherosclerosis and metabolic inflammation. Oxidase-mimetic NZs contribute to glucose-responsive systems by catalyzing glucose oxidation, enabling smart drug delivery platforms that release insulin or therapeutic proteins in hyperglycemic conditions. In neuroendocrine tumors, laccase-mimetic NZs facilitate hormone detection through selective oxidation, enhancing diagnostic sensitivity.

Compared to conventional endocrine therapeutics, such as hormone replacement therapies, biologics, or small molecule antioxidants, NZs offer distinct advantages, which are often interlinked in endocrine pathophysiology. For example, NZs with SOD-like activity reduce mitochondrial ROS in insulin-resistant tissues, restoring redox balance and enhancing insulin receptor sensitivity. Catalase-mimetic NZs further detoxify H_2_O_2_, promoting cellular survival and tissue regeneration, particularly relevant in diabetic wound healing and thyroid dysfunction. Beyond redox modulation, NZs can influence hormone biosynthesis and receptor dynamics. CeO₂ and Fe₃O₄ NZs have been shown to activate AMPK and PPARγ pathways, which regulate glucose and lipid metabolism and intersect with hormonal feedback loops. The structural tunability of NZs enables integration into smart delivery platforms (e.g., hydrogels, microneedles, mesoporous carriers) and theranostic systems that combine diagnostic and therapeutic capabilities. Through advanced surface engineering strategies, NZs also overcome biological barriers. PEGylation or ligand conjugation improves circulation time and facilitates tissue-specific accumulation, while macrophage membrane coating enables immune evasion and targeted delivery to inflamed or hormonally active tissues. Organelle-level targeting, such as lysosomal localization of Fe₃O₄ NZs, enhances intracellular signaling cascades, improving glucose uptake and insulin sensitivity. These mechanistic features position NZs as versatile agents capable of addressing the systemic complexity of endocrine disorders through catalytic precision and spatial control. The advantages of NZs also lie in their multi-functionality, engineering flexibility, and stability under physiological conditions, which surpass many traditional antioxidants and biologics. Their biomimetic and targeted delivery capabilities provide an innovative platform for intervening in complex disease networks characterized by oxidative stress and chronic inflammation. However, despite the growing body of evidence supporting the potential of NZs in endocrine and metabolic disorders, it is important to emphasize that the vast majority of these applications remain at the preclinical stage. Current findings are largely derived from *in vitro* experiments and small-animal studies, with no progression toward large-animal validation or human clinical trials. To date, no NZ-based platform targeting endocrine diseases has advanced beyond early translational research.

Several critical gaps must be addressed to facilitate clinical implementation. First, there is a need for comprehensive biosafety and toxicological profiling, particularly regarding the long-term biodistribution, clearance, and potential accumulation of metal-based nanozymes. Second, challenges in manufacturing and standardization persist, as reproducible large-scale synthesis with consistent catalytic performance remains underdeveloped. Third, the absence of a clear regulatory framework for multifunctional, enzyme-mimetic nanomaterials introduces uncertainty in approval pathways, slowing clinical translation. Fourth, most preclinical studies rely on simplified or reductionist models that inadequately replicate the complexity of human endocrine pathophysiology. Incorporation of advanced disease models, including patient-derived systems and organ-on-chip technologies, will be essential to bridge this gap. Finally, clinical integration strategies must be considered early, including how NZ-based sensors or therapeutics can complement existing diagnostic workflows and endocrine treatments. Integrating precision-targeting strategies, smart responsive designs, and combinatorial therapies with NZs may overcome current limitations and accelerate their application in metabolic disease management 140. Continued interdisciplinary research and early-phase clinical studies will be crucial to fully harness the potential of NZs as next-generation therapeutics in atherosclerosis, obesity, and their interlinked complications 141-142.

NZs pose regulatory challenges due to their hybrid nature, combining nanostructural properties with enzyme-like biochemical activity. Both the FDA and EMA classify products based on their primary mode of action and intended use. According to FDA guidance, NZs that exert their therapeutic effect through chemical action within or on the body are typically classified as drugs. If the effect is mechanical or physical, such as in diagnostic or structural applications, they may be regulated as medical devices 143. In cases where both mechanisms are present, NZs may be designated as combination products, requiring coordinated review across regulatory centers. The EMA applies a similar approach, evaluating NZs under either medicinal product or medical device legislation, depending on their mechanism of action and composition. EMA guidance on nanotechnology-based medicinal products highlights the need for case-by-case assessment, especially for borderline technologies 144. The lack of harmonized definitions and regulatory pathways for NZs complicates their clinical translation. The classification directly affects the design of preclinical studies, the required physicochemical characterization, and the scope of safety and efficacy evaluations 145.

Taken together, while NZs represent a highly versatile and innovative class of materials for endocrine medicine, their translation from laboratory to clinic will require coordinated efforts in toxicology, materials science, regulatory policy, and clinical trial design. Addressing these barriers will be crucial to realizing their full potential as next-generation diagnostic and therapeutic agents.

## 9. Conclusion and future perspectives

The convergence of nanotechnology and enzyme-mimetic systems has ushered in a transformative era for biomedical applications. NZs have demonstrated exceptional versatility across a wide range of diagnostic, therapeutic, and theragnostic domains. Their ability to modulate oxidative stress, inflammation, and metabolic dysfunctions has positioned them as potent tools in the management of *diabetes mellitus*, atherosclerosis, obesity, and other interlinked endocrine disorders. Moreover, their adaptability for incorporation into biosensing platforms, smart drug delivery systems, and regenerative medicine approaches underscores their role as next-generation biomedical agents.

In diabetes care, NZs have shown promise in non-invasive glucose monitoring and modulation of insulin resistance. Their incorporation into responsive hydrogels, microneedles, and smart therapeutics has further advanced the field of diabetic foot ulcer management. Likewise, their targeted capabilities have made significant strides in atherosclerosis by enabling plaque-specific imaging and immunomodulation. In obesity and other endocrine-related pathologies, including thyroid and adrenal disorders, NZs provide a highly adaptable and bioactive framework to intervene in complex metabolic cascades.

Despite these advancements, challenges remain. The long-term biosafety, biocompatibility, and immunogenicity of NZs *in vivo* require comprehensive evaluation. Issues such as large-scale production, reproducibility, regulatory approval, and targeted delivery mechanisms must be addressed before clinical translation can be fully realized. The potential toxicity of metal-based NZs and limitations in catalytic specificity also necessitate more refined design strategies.

### Materials engineering

To move beyond proof-of-concept, NZs must evolve from static catalysts to programmable systems capable of responding to biochemical stimuli such as ROS levels, glucose fluctuations, or hormonal gradients. This shift demands architectures with spatial and temporal control, including organelle-targeted designs and artificial intelligence-guided synthesis strategies. These innovations will enable NZs to operate with greater precision and reduced off-target effects in endocrine tissues.

### Targeting specific diseases

To maximize clinical relevance, NZs should be tailored to the molecular pathology of specific endocrine disorders. In thyroid cancer, they may remodel hypoxic microenvironments while delivering targeted therapies. In adrenal hyperfunction, cortisol-sensitive NZs could enable feedback-controlled drug release. In obesity and metabolic syndrome, NZs that modulate macrophage polarization and mitochondrial function may restore metabolic balance. These approaches require integration with hormonal feedback loops and tissue-specific signaling networks.

Looking ahead, the integration of NZs with machine learning, artificial intelligence (AI), and precision medicine offers a powerful route to optimize their design and application. Recently, AI-driven platforms such as AI-ZYMES and machine learning models are revolutionizing the rational design of NZ by predicting catalytic activity, guiding synthesis, and enabling multiplexed sensing 146-148. These approaches facilitate the development of hybrid and stimulus-responsive NZ capable of performing multiplexed tasks within complex biological systems 149-151. Future research should prioritize not only these advanced designs but also the advancement of clinical trials and interdisciplinary collaborative studies, which are essential to transition nanozyme-based technologies from bench to bedside.

### Challenges in clinical translation

Clinical translation remains the most critical hurdle. Most NZs studied are confined to *in vitro* or small-animal models, which fail to capture the complexity of human endocrine physiology. Advanced platforms such as organ-on-chip systems and patient-derived organoids will be essential to simulate hormonal crosstalk and tissue-specific responses. Early-phase human trials should focus on biosafety, biodistribution, and pharmacokinetics, especially for metal-based NZs. Embedding NZs into wearable biosensors or diagnostic workflows may facilitate clinical adoption. Regulatory frameworks must evolve to accommodate their hybrid nature, requiring coordinated evaluation across drug and device categories.

To enable clinical translation, strategies for validation that may be helpful include conducting pilot human studies utilizing NZ-based biosensors for the monitoring of glucose or endocrine biomarkers; integrating NZ platforms into established diagnostic workflows to assess their compatibility, analytical performance, and added clinical value; and performing longitudinal safety evaluations in large-animal models to investigate biodistribution, clearance kinetics, and immunogenicity under physiologically relevant conditions. In summary, NZs stand at the frontier of personalized and systems-level medicine, offering dynamic and multifunctional solutions for the detection, monitoring, and treatment of endocrine and metabolic disorders. While current evidence is promising, further refinement in design, targeting, and validation is required to ensure robust clinical applicability. As the field matures, NZs are expected to redefine therapeutic paradigms and pave the way for smarter and more integrated healthcare interventions.

## Figures and Tables

**Figure 2:**
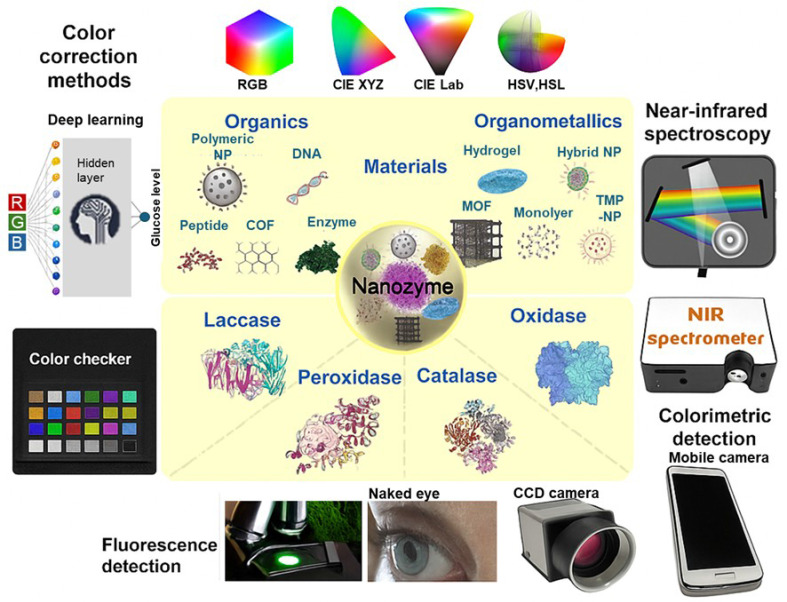
**Nanozyme-based colorimetric biosensors for glucose detection and diabetes management.** Schematic overview of nanozyme materials—classified as organic or organometallic—and their associated enzyme-mimicking activities (laccase, peroxidase, catalase, oxidase), detection methods (fluorescence, colorimetry, NIR spectroscopy), and color correction strategies using deep learning and color space model. Reproduced from Jeon et al. 35. This is an open-access article distributed under the terms of the Creative Commons Attribution License (https://creativecommons.org/licenses/by/4.0/).

**Schema 1:**
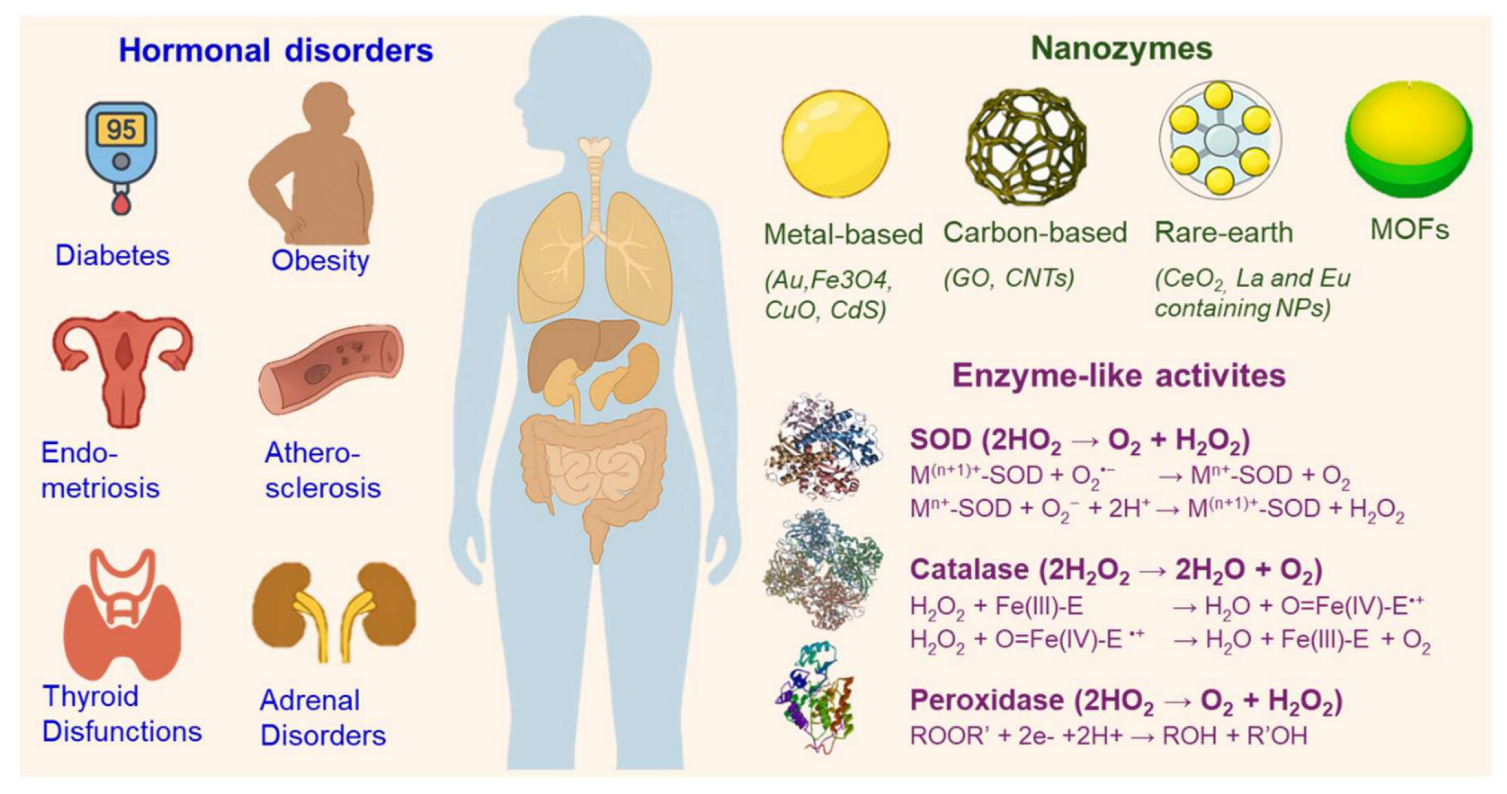
Intersection of Nanozyme Structures and Activities in Diabetes and Endocrine Disorders. SOD: Superoxide dismutase.

**Table 1:** Catalytic Activity, Advantages, and Limitations of Nanozyme Types

Nanozyme class	Dominant catalytic activities	Application advantages	Limitations or risks
Metal-based	Fe₃O₄: peroxidase-like (Fenton/Fenton-like); Au: oxidase-like; CeO₂: catalase- and SOD-like (Ce³⁺/Ce⁴⁺ redox); CuO: oxidase and peroxidase; noble metals (Pt/Pd/Ru): strong peroxidase-like	High catalytic turnover; robust redox cycling; straightforward surface functionalization; strong optical/electronic features for biosensing and imaging	Potential metal ion release; off-target ROS generation; long-term tissue accumulation; pH-dependent; batch-to-batch heterogeneity in synthesis
Carbon-based (graphene oxide, carbon nanotubes, carbon dots)	Peroxidase- and oxidase-like via defect/edge sites and π-electron networks	Large surface area and conductivity enhance electron transfer; easy hybridization with biomolecules/electrodes; good signal amplification for electrochemical or colorimetric sensing	Possible heteroatom/impurity variability; biocompatibility depends on surface chemistry; catalytic activity can be modest without doping or defects
Metal sulfide-based (CdS, MoS)	CdS: light-driven photocatalysis generating ROS; MoS₂: intrinsic peroxidase-like; some show photo-enhanced peroxidase	Semiconducting band structures enable controllable, light-activated therapy; 2D morphologies favor high active-site exposure	Phototoxic or ion toxicity concerns; may require light delivery and O_2_; stability and oxidative leaching under physiological conditions
Rare-earth element-based (Ce, La, Eu)	CeO₂: catalase/SOD-like; La-based: redox/electron-transfer catalysis; Eu-based: luminescent probes with auxiliary catalytic roles	Intrinsic antioxidant mimicry (redox buffering) plus strong, tunable luminescence for imaging/theragnostic platforms	Valence-state control is sensitive to environment; dopant alters activity; potential long-term retention; unclear clearance profiles
Hybrid/Composite (MOFs, core-shell, NP@MOF)	Multi-enzyme cascades (e.g., GOx & peroxidase/catalase in one construct); porous frameworks can host catalytic centers	Programmable porosity and multivalent active sites; co-loading of drugs/enzymes for synergistic therapy; improved stability and targeting via shells	Framework stability in biological fluids varies; metal nodes and ligand can contribute to toxicity; synthesis scalability and standardization remain challenging

GOx: Glucose oxidase; MOFs: Metal-organic frameworks; ROS: Reactive oxygen species; SOD: Superoxide dismutase

**Table 2:** Overview of Nanozyme Compositions, Enzymatic Activities, and Biological Effects in Diabetic Wound Healing.

Nanozyme	Base	Nanozyme activity	Biological effects	Animal model	Ref.
Mn CoO MOFs	Hydrogel	Catalase	Macrophage polarization, epithelialization, collagen deposition, angiogenesis.	Diabetic rats	53
Au MoS_2_	Hydrogel	Glucose oxidase Peroxidase	Anti-inflammatory, antibacterial	Diabetic rats	54
Au Porphyrin MOFs		ROS generation, photothermal.	Upregulation of cell proliferation factors, antibacterial.	Diabetic rats	55
MnO_2_	Hydrogel Pravastatin	ROS-scavenging, O_2_ generation	Decreased neutrophil infiltration. Macrophage polarization. Reduction of IL-1β, IL-6, TNF-α, CXCL-1. Increase of IL-4 and IL-10, TGF-β Neovascularization. Collagen deposition. Antibacterial	Diabetic rats and mice	52
Au-Pt	Hydrogel	Glucose oxidase, Catalase	Reduction of ROS damage. Antibacterial	Diabetic rats	56
Fe_3_O_4_	Glucose oxidase	(pH-switchable) Glucose oxidase Catalase Peroxidase	Proliferation and remodeling	Diabetic mice	57
Aptamer-functionalized NZ	Glucose oxidase Hyaluronic acid	Antibacterial (activated by bacteria-secreted hyaluronidase)	Antibacterial	Diabetic mice	58
Fe_2_(MoO_4_)_3_	Glucose oxidase	Catalase	Reduction of ROS damage Antibacterial	Diabetic mice	59
Prussian blue NZ	Microneedle Vascular Endothelial Growth Factor, Polymixin	Antioxidant Antibacterial	Proangiogenesis Antibacterial	Diabetic mice	60
Pt	Hydrogel	Glucose oxidase NADH oxidase Peroxidase Catalase SOD	Anti-inflammatory Proliferation Antibacterial	Diabetic rats	61
Au-Cu_2_ MoS_4_	Microneedle	Glucose oxidase Catalase	Antibacterial	Diabetic mice	62
Cu Zr MOFs	Hydrogel	ROS scavenging	Anti-inflammatory Proliferation	Diabetic mice and rats	63
MnO_2_	Fiber-based compartmentalization	Peroxidase, catalase	Anti-inflammatory Proliferation	Diabetic mice	64
PtCuTe	-	ROS scavenging Antibacterial	Vascular formation, macrophage polarization, fibroblast mobility, regeneration of highly vascularized skin. Antibacterial.	Diabetic mice	65
Pt	hydrogel	Glutathione reductase Catalase Peroxidase	Anti-inflammatory Angiogenesis	Diabetic mice	66
Cu	2,5-dimercaptoterephthalic acid	Catalase	Cell proliferation, migration, angiogenesis, antibacterial	Diabetic mice	67
MnO_2_	Bioactive glasses, cryogel	ROS scavenging Peroxidase	Cell proliferation, ant-inflammatory, collagen III deposition, angiogenesis, antibacterial	Diabetic rats	68
Au, CuO	Hydrogel	SOD, catalase. glucose oxidase, peroxidase, nitric oxide synthase	Lower glucose, angiogenesis, antibacterial	Diabetic rats	69
Fe_2_C	Glucose oxidase. Microneedle	Antibacterial	Antibacterial	Diabetic mice	70
Fe, Au	-	Glucose oxidase, Peroxidase Antibacterial	Lower glucose, Antibacterial	Diabetic mice	71
CoO	Hydrogel	Catalase	Anti-inflammatory, increased re-epithelialization, collagen deposition, functional blood vessel growth	Diabetic rats	72
Se	Janus liposozyme	ROS scavenging	Macrophage polarization, skin wound repair. Tissue regeneration in mini pigs. Antibacterial	Diabetic mice and mini pigs	51
FeS Au	H_2_S	Glucose oxidase Peroxidase	Upregulation of HIF-1, VEGF, and angiogenesis factors. Protection of endothelial cells. Angiogenesis, collagen deposition Antibacterial.	Diabetic rats	73
MOFs	Zr Natural SOD	ROS scavenging	Inhibition of fibroblast senescence, ferroptosis, and STING signaling pathway activation in macrophages.	Diabetic mice	74
CeO_2_	Glucose oxidase	Peroxidase Catalase	Angiogenesis, anti-inflammatory, antibacterial.	Diabetic mice	75
Cu ZnO	Hydrogel Rhein	SOD Antibacterial	Vascular regeneration, collagen deposition, anti-inflammatory, antibacterial.	Diabetic rats	76
Ru	Hydrogel	Nitric oxide and ROS scavenging	Bone macrophage polarization, improved bone marrow-derived mesenchymal stem cells, and endothelial cells	Diabetic mice	77
Zn-based polymetallic oxonate	Aldehyde and methacrylic anhydride-modified hyaluronic acid hydrogel, chitosan nanoparticles, glucose oxidase	Catalase, SOD	macrophage polarization through MAPK/IL-17, lower pro-inflammatory cytokines, higher anti-inflammatory mediators, angiogenesis, collagen regeneration. Antibacterial.	Diabetic rats	78
Os	Hydrogel Glucose oxidase	Catalase, hydroxyl free radicals' generation	Lower bone inflammation, lower glucose levels, antibacterial	Diabetic mice	79
Cu	Polyphenol ligands	ROS scavenging	Re-epithelialization, collagen deposition, angiogenesis, and immunoregulation	Diabetic rats	80
Fe	Hydrogel	Catalase	Epidermis formation and collagen deposition	Diabetic mice	81
Prussian blue NZ	Zr	ROS scavenging	Macrophage polarization, activation of vascular endothelial cells.	Diabetic rats	82
Au@Pt	Hydrogel	Glucose Oxidase Catalase	Bone marrow mesenchymal stem cells protection. Osteogenic differentiation. Anti-inflammatory. Angiogenesis. Improved bone regeneration.	Diabetic rats	83
Zn	C-dots	ROS scavenging	Anti-inflammatory, angiogenesis, collagen deposition, tissue remodeling. Antibacterial.	Diabetic rats	84
Cu CeO_2_	Hydrogel JSH-23	ROS scavenging	Improved Nrf2 transcriptional activity of macrophages.	Diabetic mice	85
Au Prussian blue	Not specified	Peroxidase	Upregulation of VEGF and CD31. Angiogenesis. Antibacterial	Diabetic rats	86
MnO₂ Au MOFs	Hydrogel mSiO_2_ Acidic fibroblast growth factor	Catalase SOD	Cell proliferation, migration, angiogenesis macrophage polarization	Diabetic mice and rats	87
Au Pt	Hydrogel DNA aptamer	Glucose oxidase ROS scavenging	Anti-inflammatory Antibacterial	Diabetic mice	88
Cu Se	Hydrogel Glucose oxidase	Peroxidase	Production of mature collagen. Migration and proliferation of endothelial cells Antibacterial	Diabetic rats	89
MnO_2_	Hydrogel	ROS scavenging	Fibroblast proliferation Angiogenesis. Antibacterial	Diabetic mice and rats	90
Fe_3_O_4_	Glucose oxidase l-arginine (l-Arg)	Catalase NO release	Antiinflammatory Antibacterial	Diabetic mice	91
Cu	Lysyl oxidase (catalytic domain)	Lysyl oxidase	Bone collagen synthesis and biomineralization.	Diabetic mice	92
Fe Cu	Tannic acid	Peroxidase	Vascular migration and regeneration Antibacterial	Diabetic mice	93
Fe_3_O_4_	MXene microneedle	Catalase SOD	ROS upregulation, lipid peroxidation.	Diabetic rats	94
CuP	Hydrogel	Peroxidase catalase	Angiogenesis. Antibacterial	Diabetic mice	95
Mo Fe/Cu	Hydrogel Glucose oxidase	Peroxidase Catalase SOD	Angiogenesis. Antibacterial	Diabetic mice	96
Ni MOFs	-	ROS scavenging	Cell migration, angiogenesis, TGF-β1 activation, macrophage polarization	Diabetic mice	97
Au Pd	Hydrogel	Glucose oxidase Catalase SOD	NF-κB inhibition, macrophage polarization, cell migration, and vascularization.	Diabetic mice	98
Pt	Hydrogel	Catalase	Granulation tissue formation, angiogenesis, type III collagen deposition. Antibacterial	Diabetic mice	99
Cu	-	ROS scavenging	Antiinflammatory, collagen synthesis, angiogenesis	Diabetic mice	100
Cu, O, Zn, MOFs	Hydrogel	Catalase	Macrophage polarization, cell migration, angiogenesis, collagen deposition	Diabetic rats	101
Cu MOFs	Hydrogel Glucose oxidase	Catalase	Angiogenesis, collagen deposition. Antibacterial	Diabetic rats	102
FeS	Aerogel Glucose oxidase	Glucose oxidase	Antibacterial.	Diabetic mice	103

CD31: cluster of differentiation 31; CXCL-1: chemokine (C-X-C motif) ligand 1; HIF-1: Hypoxia-Inducible Factor 1; IL: interleukin; MAPK: Mitogen-Activated Protein Kinase; NADH: Nicotinamide Adenine Dinucleotide (Reduced form); NF-κB: nuclear factor kappa-light-chain-enhancer of activated B cells; NO: nitric oxide; Nrf2: nuclear factor erythroid 2-related factor 2; ROS: reactive oxygen species; SOD: superoxide dismutase; STING: stimulator of interferon genes; TGF-β1: transforming growth factor beta 1; TNF: tumor necrosis factor; VEGF: vascular endothelial growth factor.

**Table 3:** Summary of nanozyme-based therapeutic strategies for non-diabetic endocrine disorders.

Target	Nanozyme Type	Function	Detection Method	*In vitro/in vivo*	Key Outcome	Ref.
Adrenal/Cortisol	Au	HRP-like, signal amplification	Electrochemical	*In vitro* diagnostic	Ultra-sensitive cortisol detection	119
Adrenal/Cortisol	AuNPs@SnS_2_/NiCo MOFs	electrochemical		*In vitro* diagnostic	Ultra-sensitive cortisol detection	120
Adrenal/Cortisol	Ag, Cd, Au	photoelectrochemical		*In vitro* diagnostic	Ultra-sensitive cortisol detection	121
Methimazole / thyroid drug	Fe	peroxidase	Colorimetric	*In vitro* diagnostic	Linear range of 5-50 mM	123
Thyroid	Cu	peroxidase	-	*In vitro* diagnostic	drug discovery	124
Thyroid cancer	Pt	ROS generation	-	*In vivo* (mice)	Cancer cell apoptosis	125
Adrenal/Catecholamines	Au@CuMOF	GOx- and peroxidase-like	POC microfluidic + PGM	*In vitro* diagnostic	Multiplex CTC detection	126
Adrenal/Catecholamines	N-doped Co/CoOx	Laccase-like	Colorimetric	*In vitro* diagnostic	Sensitive epinephrine detection	127
Adrenal/Catecholamines	Cu-CP	Laccase-like	Fluorescence + Colorimetric	*In vitro* diagnostic	Sequential dual biomarker detection	128
Adrenal/Catecholamines	COF@Pt	Peroxidase mimic	Electrochemical +colorimetric	*In vitro* diagnostic	High sensitivity	129
Adrenal/Catecholamines	CuCoFe-LDH	Laccase mimic	Smartphone colorimetric	*In vitro* diagnostic	Visual detection	130
hCG	Pd@Pt-Ru	Peroxidase-like	LFIA		High sensitivity	131
hCG	CO-Pd@Pt	Peroxidase-like	LFIA	*In vitro* diagnostic	High sensitivity	132
hCG	Au/Fe3O4	Peroxidase-like	ICT strip	*In vitro* diagnostic	High sensitivity	133
hCG	β-FeOOH	Peroxidase mimic	ICT strip	*In vitro* diagnostic	High sensitivity	134
Estradiol (Endometriosis)	MnO2	Estrogen scavenging	-	*In vivo* (mice)	Therapeutic. Suppressed lesion growth	135
Cyrcardian/Melatonin	PtRu	Peroxidase-like	Fluorescence (ratiometric)	*In vitro* diagnostic	Smartphone sensing, LOD = 23 nM	136
Gastrointestinal / Gastrin	Fe_3_O_4_@Pt	Dual-mode (magnetic & peroxidase)	Magnetic LFIA	*In vitro* diagnostic	LOD = 3 pg/mL	137

CTC: circulating tumor cells; hCG: human chorionic gonadotropin; ICT: immunochromatographic test; LFIA: lateral flow immunoassay; LOD: limit of detection; PGM: personal glucose meter; POC: point-of-care; ROS: reactive oxygen species.

**Table 4:** Nanozyme classes, catalytic mechanisms, target organs, and disease applications in endocrine and metabolic disorders.

Catalytic Mechanism	Nanozyme Class	Target Organs	Diseases
SOD-mimetic	CeO₂	Liver, Skeletal Muscle	Diabetes, Obesity, Neurodegeneration, Wound Healing
	AuCePt	Liver	Diabetes
	Au	Adipose Tissue	Obesity
	Cu/Zn	Liver, Adipose Tissue	Obesity, Alcoholic Liver Disease
	Nickel MOFs	Skin	Diabetic Wound Healing
Peroxidase-mimetic	Fe₃O₄	Liver	Diabetes
	Prussian Blue	Vascular System	Atherosclerosis
	AuPt	Liver, Gut	Diabetes
	PtSiO₂	Liver	Diabetes
	MnO₂	Skin	Endometriosis, Wound Healing
	Fe	Blood	Thyroid Drug Monitoring
	Ru	Blood	Diabetes, Cancer
Peroxidase-mimetic	Pd@Pt	Blood	hCG Detection
	Au/Fe₃O₄	Blood	hCG Detection
Oxidase-mimetic	Au	Blood	Glucose Detection
	CuO	Skin	Wound Healing
	RuN	Blood	Diabetes, Cancer
	Pt	Thyroid	Thyroid Cancer
	Pt-Ru	Blood	Melatonin Detection
Catalase-mimetic	CeO₂	Liver, Skeletal Muscle	Diabetes, Obesity, Neurodegeneration
	AuCe	Liver	Diabetes
	Pt-SiO₂	Liver	Diabetes
	CeO₂	Blood	Diabetes
Laccase-mimetic	CuCoFe	Blood, Urine	Pheochromocytoma
	Co/CoOx	Blood	Pheochromocytoma
	Cu	Urine	Pheochromocytoma
